# Long-chain unsaturated free fatty acids reduce the host cell invasion of *Listeria monocytogenes* outbreak strains

**DOI:** 10.3389/fcimb.2025.1542165

**Published:** 2025-02-28

**Authors:** Caroline Borreby, Thea Amalie Hvidtfeldt, Magnus Ganer Jespersen, Patricia T. dos Santos, Sofie Dam Houborg, Eva Maria Sternkopf Lillebæk, Michael Kemp, Birgitte H. Kallipolitis

**Affiliations:** ^1^ Department of Biochemistry and Molecular Biology, University of Southern Denmark, Odense, Denmark; ^2^ Department of Infection and Immunology at The Peter Doherty Institute for Infection and Immunity, University of Melbourne, Melbourne, VIC, Australia; ^3^ The Regional Department of Clinical Microbiology, Zealand University Hospital - Koege, Koege, Denmark; ^4^ Department of Clinical Medicine, University of Copenhagen, Copenhagen, Denmark

**Keywords:** *Listeria monocytogenes*, PrfA, antimicrobial, anti-virulence, free fatty acids

## Abstract

The Gram-positive bacterium *Listeria monocytogenes* is a highly adaptable pathogen capable of causing severe foodborne infections, particularly in vulnerable populations. During infection, *L. monocytogenes* uses a variety of virulence factors to invade and multiply within host cells. The transcriptional regulator PrfA controls the expression of these virulence factors and is essential for the intracellular lifestyle of *L. monocytogenes*. Long-chain unsaturated free fatty acids (FFAs) have long been recognized for their antimicrobial activity and were recently shown to inhibit PrfA-dependent virulence gene expression in *L. monocytogenes*. To date, the antimicrobial and anti-virulent activities of FFAs have been primarily studied in laboratory strains. However, to fully evaluate their potential as anti-infective agents, it is essential to assess the effects of long-chain FFAs on clinically relevant isolates, including outbreak strains associated with high-fat food products. Here, we demonstrate that five different clinically relevant *L. monocytogenes* isolates are sensitive to the antimicrobial activity of long-chain unsaturated FFAs. Furthermore, at subinhibitory concentrations, these FFAs inhibit PrfA-regulated expression of virulence factors across all tested strains and reduce their invasive potential in non-phagocytic cells. These findings underscore the potential of long-chain unsaturated FFAs in developing new preventive strategies against *L. monocytogenes* strains associated with severe foodborne infections.

## Introduction

*Listeria monocytogenes* is a Gram-positive, facultative intracellular pathogen and the causative agent of the severe disease listeriosis (Freitag et al., 2009; Radoshevich and Cossart, 2018; Koopmans et al., 2023). The bacterium is ubiquitous in the environment and is frequently isolated from water and soil. Because of its ability to withstand harsh conditions − including a wide range of temperatures, pH levels, and salt concentrations − *L. monocytogenes* is able to persist in food production environments and can be found in many food sources (Swaminathan and Gerner-Smidt, 2007; Radoshevich and Cossart, 2018; Koopmans et al., 2023). Frequently contaminated foods include dairy products, vegetables, ready-to-eat products, delicatessen meats, and cold-smoked fish, amongst others. Consequently, infections with *L. monocytogenes* are primarily foodborne, following the ingestion of contaminated foods (Posfay-Barbe and Wald, 2009; Radoshevich and Cossart, 2018; Koopmans et al., 2023).

Although human listeriosis cases generally occur sporadically, large-scale foodborne outbreaks frequently emerge, making *L. monocytogenes* a significant threat to global health (Koopmans et al., 2023). In healthy individuals, *L. monocytogenes* infections may result in mild symptoms, such as gastroenteritis, while more severe complications can develop in high-risk groups, including the elderly, immunocompromised individuals, pregnant women, neonates, and young children (Radoshevich and Cossart, 2018; Koopmans et al., 2023). In susceptible individuals, *L. monocytogenes* may cross internal barriers, such as the intestinal barrier, blood-brain barrier, and the placental barrier. Consequently, infections caused by *L. monocytogenes* can manifest as bacteremia, septicemia, meningitis, or pregnancy-associated listeriosis, with a case-fatality rate of approximately 20-30%, even with conventional antibiotic treatment (Radoshevich and Cossart, 2018; Koopmans et al., 2023).

As a multifaceted human pathogen, *L. monocytogenes* can infect both non-phagocytic and phagocytic cells by promoting the expression of key virulence factors (Freitag et al., 2009; Radoshevich and Cossart, 2018; Koopmans et al., 2023). These factors enable the bacterium to invade, multiply, and spread within the host. Initially, *L. monocytogenes* induces internalization into non-phagocytic host cells, facilitated by two key virulence factors: internalin A (InlA) and internalin B (InlB) (Lecuit et al., 2001). Once inside the cell, the pathogen must escape from the vacuole to avoid degradation. To this end, *L. monocytogenes* utilizes the pore-forming toxin listeriolysin O (LLO) and two phospholipases, PlcA and PlcB, to effectively release itself into the host cell cytosol (Schnupf and Portnoy, 2007). The cytosol provides a more favorable environment for the bacterium, where rapid proliferation is supported by the hexose phosphate transporter Hpt (Chico-Calero et al., 2002). Finally, *L. monocytogenes* promotes cell-to-cell spread, avoiding exposure to the extracellular environment, through the formation of an actin comet tail mediated by the virulence factor Actin assembly-inducing protein (ActA) (Freitag et al., 2009; Posfay-Barbe and Wald, 2009; Radoshevich and Cossart, 2018; Koopmans et al., 2023). Importantly, the genes encoding these key virulence factors are all regulated by PrfA, the master transcriptional regulator of virulence in *L. monocytogenes*. PrfA integrates various environmental cues that signal the transition of *L. monocytogenes* from a harmless saprophyte to a human pathogen. Therefore, PrfA and PrfA-regulated virulence genes are essential for the pathogenesis of *L. monocytogenes* (Scortti et al., 2007; Freitag et al., 2009).

In our previous work, we demonstrated that naturally occurring long-chain unsaturated free fatty acids (FFAs) exert antimicrobial and/or anti-virulent activities against *L. monocytogenes* (Sternkopf Lillebaek et al., 2017; Dos Santos et al., 2020; Thomasen et al., 2022a). At higher concentrations, long-chain polyunsaturated FFAs were shown to inhibit bacterial growth, potentially by compromising the integrity of the bacterial membrane (Borreby et al., 2023). A laboratory strain lacking *prfA* displayed increased tolerance to FFAs, suggesting a sensitizing role for PrfA in the response of *L. monocytogenes* to antimicrobial FFAs. Furthermore, at lower concentrations, some long-chain unsaturated FFAs exhibit anti-virulent activities in *L. monocytogenes* laboratory strains. These FFAs act to suppress the expression of PrfA-regulated virulence genes, likely through the inhibition of PrfA´s DNA-binding activity (Sternkopf Lillebaek et al., 2017; Dos Santos et al., 2020; Thomasen et al., 2022a).

As noted above, *L. monocytogenes* is frequently found to contaminate high-fat food products, such as smoked fish and delicatessen meats, which consequently serve as sources of listeriosis outbreaks. These findings make it relevant to investigate how *L. monocytogenes* outbreak strains originating from contaminated high-fat foods respond to long-chain FFAs. In this study, we aimed to determine whether such isolates are particularly well-adapted to resist the antimicrobial and/or anti-virulence activities of long-chain unsaturated FFAs. To this end, we selected three isolates of sequence types 6, 8, and 391 (ST6, ST8, and ST391) derived from listeriosis outbreaks in Denmark linked to cold-smoked or cured salmon (Gillesberg Lassen et al., 2016; Schjorring et al., 2017). We also included an outbreak strain, ST224, isolated from a large-scale Danish outbreak in 2014 originating from spiced meat rolls, another high-fat food product (Kvistholm Jensen et al., 2016). Finally, we included an *L. monocytogenes* isolate, ST2, which has been linked to severe invasive diseases, such as central nervous system (CNS) infections and pregnancy-associated listeriosis (Jensen et al., 2016). We confirmed the presence of PrfA-regulated virulence genes in these strains, and we analyzed the expression of virulence genes in response to long-chain FFAs. Furthermore, we investigated whether long-chain FFAs affect the infection of cell lines by these clinically relevant *L. monocytogenes* isolates. We found that at high concentrations, long-chain polyunsaturated FFAs inhibit the growth of all strains tested. Importantly, subinhibitory concentrations of long-chain unsaturated FFAs inhibit the PrfA-regulated expression of virulence factors and interfere with the invasive potential of the *L. monocytogenes* outbreaks strains. Overall, our findings confirmed that clinically relevant strains of *L. monocytogenes* are sensitive to the antimicrobial and anti-virulence activities of long-chain unsaturated FFAs.

## Materials and methods

### Bacterial strains and growth conditions

The strains used in this study were *L. monocytogenes* EGD-e serovar type 1/2a (CC9, lineage II, ATCC BAA-679), *L. innocua* Clip11262 (CIP 107775), and five clinically relevant *L. monocytogenes* isolates, kindly provided by Eva Møller Nielsen, Head of Section for Foodborne Infections, Statens Serum Institut, Denmark. Of the clinically relevant strains, three were lineage I strains, which are often associated with severe clinical cases and large-scale listeriosis outbreaks worldwide (Jensen et al., 2016; Maury et al., 2016): ST2 (isolate number 20071038, CC2, serovar 4b), ST6 (isolate number 20140930, CC6, serovar 4b), and ST224 (isolate number 20141085, CC244, serovar 2b). ST8 (isolate number 20171866, CC8, serovar 2a) and ST391 (isolate number 20141037, CC89, serovar 2a) were lineage II strains, which are typically associated with less severe cases (Orsi et al., 2011). Strains of *L. monocytogenes* were routinely grown in brain heart infusion medium (BHI; Oxoid) at 37°C with aeration, unless otherwise specified. When required, BHI was supplemented with 1% Amberlite(R) XAD-4 (Merck).

### Free fatty acids

The following FFAs were used in this study: eicosapentaenoic acid (EPA; C20:5^5,8,11,14,17^ *cis*; purity ≥ 99%), γ-linolenic acid (GLA; C18:3^6,9,12^, *cis*; purity ≥ 99%), linoleic acid (LNA; C18:2^9,12^ *cis*; purity ≥ 99%), oleic acid (OA; C18:1^9^, *cis* purity ≥ 99%), and stearic acid (SA; C18:0; purity ≥ 99%). All FFAs were obtained from Sigma-Aldrich. All FFAs were solubilized in 96% ethanol as the vehicle. The saturated FFA (SA) was stored at room temperature, while unsaturated FFAs (EPA, GLA, LNA, and OA) were stored at –20°C under nitrogen gas to prevent oxidation.

### Whole-genome sequencing

Bacterial isolates were cultured on 5% blood agar (SSI Diagnostica). DNA was extracted using PowerBead tubes (Qiagen) and purified with AMPure XP beads (Beckman Coulter) and ethanol washes. DNA concentration was assessed using the Qubit dsDNA Broad Range assay (Thermo Fisher Scientific). DNA libraries were prepared according to the Native SQK-LSK109 protocol from Oxford Nanopore Technologies with additional reagents from New England Biolabs (Cat. E7564, M0367, and E6056S). The ONT sequencing was run on a GridION (Oxford Nanopore Technologies) with FLO-MIN104/R9.4.1 flow cells.

For Illumina sequencing of the bacterial isolates, we refer to (Gillesberg Lassen et al., 2016; Jensen et al., 2016; Kvistholm Jensen et al., 2016; Schjorring et al., 2017).

Illumina reads were filtered and trimmed with TrimGalore v.0.6.4. Nanopore reads were filtered with NanoFilt v.2.8.0 (De Coster et al., 2018). Hybrid assembly was done using Unicycler v. 0.4.9b with default settings (Wick et al., 2017).

### *In silico* screen and sequence identity

Genes were screened using Abricate v.0.9.9 [Seemann T, Abricate, Github https://github.com/tseemann/abricate] and the virulence finder database (Chen et al., 2016). Sequence identities were calculated using blastn or blastp, using all-v-all comparisons with blast+ v.2.11.0. Sequence identities were plotted using R v.4.1.1 and the pheatmap package v.1.0.12. Single nucleotide polymorphisms (SNPs) were called from alignments using SNP-sites (Page et al., 2016).

### Growth experiments in 96-well plates

For growth experiments in culture flasks, overnight (ON) cultures of EGD-e, ST2, ST6, ST8, ST224, and ST391 were diluted to optical density at wavelength 600 (OD_600_) = 0.002 in BHI. Culture flasks were incubated at 37°C with aeration. Growth was monitored by OD_600_ measurements until stationary phase had been reached for all strains. Growth experiments with different stressing agents (ampicillin, gentamicin, or ethanol) were conducted in a plate reader (Synergy ™ H1 multi-mode microplate reader, BioTek) using 96-well plates (SARSTEDT). ON-cultures of EGD-e, ST2, ST6, ST8, ST224, and ST391 were diluted to a final OD_600_ = 0.005 in the 96-well plate containing BHI supplemented with varying concentrations of the stressing agent. The 96-well plate was incubated at 37°C, with 15 seconds of orbital shaking prior to OD_600_ measurements every 30 minutes for 24 hours. The experiments were conducted in three biological replicates.

### Growth experiments in the presence of free fatty acids

For growth experiments with FFAs, ON-cultures of EGD-e, ST2, ST6, ST8, ST224, and ST391 were diluted to OD_600_ = 0.0002 in 4 mL BHI in glass culture tubes. Cultures were subjected to increasing concentrations of either EPA (5-30 µg/mL EPA), GLA, LNA, OA, or SA (30-90 µg/mL). Cultures were incubated at 37°C with aeration for 20 hours, and growth was subsequently assessed by measuring OD_600_. The inhibitory concentration (IC) of each specific FFA was defined as the lowest concentration resulting in growth inhibition (OD_600_ ≤ 0.1) (Sternkopf Lillebaek et al., 2017; Dos Santos et al., 2020). To account for precipitation of saturated FFAs, controls containing BHI with 30-90 µg/mL SA were included. Untreated controls and vehicle-treated controls were also included in these experiments. All experiments were performed in three biological replicates.

### Preparation of total RNA samples and northern blot analysis

ON-cultures of selected strains were diluted to OD_600_ = 0.02 in BHI supplemented with 1% Amberlite XAD-4 and grown until OD_600_ = 0.35. Cultures were then split and treated with subinhibitory concentrations of EPA (2 µg/mL), or GLA (2 µg/mL), LNA, OA, or SA (3 µg/mL). Vehicle-treated samples were included as controls for all experiments.

15 mL culture was harvested from FFA-treated and vehicle-treated samples at the indicated time points (30 minutes to 5 hours). Samples were immediately snap-cooled in liquid nitrogen and centrifuged at 8000 rpm for 3 minutes at 4°C. Total RNA was extracted using Tri reagent (Invitrogen) and cells were subsequently disrupted twice using a FastPrep^®^-24 instrument, and as previously reported in (Nielsen et al., 2010). The integrity, purity and concentration of the total RNA was confirmed by agarose gel electrophoresis and on a DeNovix DS-11 Fx+ instrument.

Agarose Northern Blots were performed as previously reported (Dos Santos et al., 2020). Membranes were hybridized with ^32^P-labeled single-stranded probes (see **Supplementary Table S1**). Bands were visualized with Typhoon FLA9000 (GE Healthcare) and subsequently quantified using IQTL 8.0 quantification software (GE Healthcare). The experiments were performed in three biological replicates; representative examples are shown. Differences were considered significant if the relative normalized RNA levels (FFA versus vehicle control) consistently fell below 0.4 or exceeded 2.5 across all replicates.

### Preparation of protein extracts and western blot analysis

ON-cultures of selected strains were diluted to OD_600_ = 0.02 in fresh BHI supplemented with 1% Amberlite XAD-4 and grown until OD_600_ = 0.35. Cultures were then split and treated with subinhibitory concentrations of EPA (final concentration of 2 µg/mL), or GLA (final concentration of 2 µg/mL), LNA, OA or SA (final concentration of 3 µg/mL). Vehicle-treated cultures were included as controls. Cultures were FFA-treated for 3 hours and subsequently 4 mL of the cultures were harvested and centrifuged at 3000 rcf for 7 minutes at 4°C. The supernatant was removed, and cell pellets were resuspended in lysis buffer (0.1 M NaCl, 50 mM Tris-HCl (pH = 7.5), 10 mM EDTA and 0.5% SDS). Preparation of protein extracts and Western Blot analysis was conducted as previously described in (Sternkopf Lillebaek et al., 2017), with some modifications: membranes were stained with Coomassie Blue staining and blotting was performed at 20 V for 45 minutes. Primary antibodies (α-LLO and α-ActA, Abnova) and the secondary antibody (Goat α-Mouse IgG (H+L), Hrp, Invitrogen) were diluted 1:3000 in TTBS + 0.3% dry-milk. All blots were developed using an Amershan ImageQuant 800 (GE Healthcare). Relative protein quantities were normalized to a representative band on the Coomassie-stained membrane using ImageJ software as described previously in (Sievers et al., 2014; Sternkopf Lillebaek et al., 2017). The experiments were conducted in three biological replicates; representative examples are shown. Differences were considered significant if the relative normalized protein levels (FFA versus vehicle control) consistently fell below 0.4 or exceeded 2.5 across all replicates.

### Mammalian cell cultures

The human colon carcinoma cell line Caco-2 (ATCC HTB-37) and J774A.1 murine macrophage cell line (ATCC TIB-67^™^) were used in this study. Caco-2 cells were cultured in Dulbecco’s Modified Eagle Medium (DMEM; Gibco) supplemented with 20% heat-inactivated fetal bovine serum (FBS; Gibco) and 1% penicillin/streptomycin (PS; Gibco; 100 µg/mL). J774A.1 cells were cultured in DMEM supplemented with 10% heat-inactivated FBS and 1% PS. Both cell lines were cultured in T75 flasks and incubated at 37°C and 5% CO_2_. The media was changed every 48-72 hours by discarding the media, washing the cells either once (for J774A.1 cell lines) or three times (for Caco-2 cell lines) in pre-heated 1 x phosphate buffered saline (PBS), and subsequently adding new, pre-heated DMEM with appropriate supplements. When either cell line reached a confluence of 70-90%, the cells were sub-cultured in an appropriate ratio. For J774A.1 cell lines, cells were washed once in 1 x PBS and subsequently DMEM with appropriate supplements was added. Cells were dislodged manually using a cell scraper and diluted into new media in an appropriate ratio and seeded into new T75 flasks. For Caco-2 cell lines, cells were washed three times in 1 x PBS and once in a 1:4 dilution of 0.25% trypsin-EDTA (Gibco) in 1 x PBS. Cells were incubated for app. 5 minutes. DMEM was added to inactivate the trypsin, and the cell suspension was centrifuged at 150 g for 5 minutes at 37°C. The supernatant was discarded, and the pellet was resuspended in an appropriate amount of DMEM with supplements and seeded into new T75 flasks.

### Invasion assays in Caco-2 cell lines

Differentiation into intestinal epithelium and expression of surface proteins was promoted by allowing Caco-2 cells to differentiate 18-21 days in collagen-coated 12-well plates (Corning; BioCoat Collagen I Multi Well Plates) prior to invasion assays. Approximately 0.45x10^6^ Caco-2 cells were seeded into each well. On the day prior to the invasion assay, the media was discarded, and the cells were incubated ON with 1 mL fresh, pre-heated DMEM only supplemented with 20% FBS (i.e., not supplemented with PS).

On the day of the experiment, ON-cultures of all strains were diluted to OD_600_ = 0.02 in fresh BHI supplemented with 1% XAD-4 and grown until OD_600_ = 0.35. Cultures were split at this stage and treated with subinhibitory concentrations of EPA (2 µg/mL), or GLA (2 µg/mL), LNA (3 µg/mL), OA (3 µg/mL), or SA (3 µg/mL). Cultures treated with the vehicle only were included as controls. Following 3 hours of either vehicle- or FFA-treatment, samples were harvested from each condition. Samples were centrifuged at 3200 x g at 4 °C for 5 minutes. The supernatants were discarded, and pellets were resuspended and washed once in 5 mL 1x PBS. The bacterial inoculum was prepared in DMEM + 20% FBS and used to infect the Caco-2 cells with a multiplicity of infection (MOI) of 100 (Rukit et al., 2022). The bacterial inoculum (input CFU/mL) was added to the wells, and the 12-well plate was centrifuged briefly to induce uptake of the bacteria. Bacterial cells were allowed to infect the Caco-2 cells for 30 minutes, while incubated at 37°C and 5% CO_2_. The bacterial inoculum was determined by plating serial dilutions of the bacterial DMEM-solution on BHI agar. Following the infection period, cells were washed twice in 1 mL DMEM + 20% FBS and incubated for 30 minutes with 1 mL DMEM + 20%FBS containing 50 µg/mL gentamicin, to eliminate extracellular bacteria. Cells were then washed twice with 1 mL 1xPBS, followed by addition of 1 mL 1xPBS+0.1% Triton X 100 to lyse the Caco-2 cells. The amount of intracellular bacterial cells was determined by plating serial dilutions on BHI agar (output CFU/mL). BHI agar plates were incubated ON at 37°C and enumerated to determine the invasion efficiency for each strain relative to EGD-e. The invasion percentage for each strain was calculated as follows:


$$Invasion\;percentage=(\frac{output}{input})\cdot 100$$

The invasion efficiency refers to the invasion percentage for a given strain (vehicle or EPA) relative to the invasion percentage for EGD-e (vehicle). All invasion experiments were performed in at least three biological replicates and statistical analysis was performed using multiple unpaired t-tests with Welch’ correction. All statistical tests were performed using GraphPad Prism. Differences were considered statistically significant at a confidence level of 95% (p < 0.05).

### Intracellular replication assays in J774A.1 cell lines

Preceding the replication assay, the J774A.1 cells were sub-cultured in an appropriate ratio in DMEM+10% FBS+1% PS and added to 24-well plates. Each well contains approximately 2.5x10^5^ J774A.1 cells. Plates were incubated at 37°C and 5% CO_2_. The day prior to the replication assay, the media from each well was discarded, and the cells were incubated ON with 500 µL pre-heated DMEM+ 10% FBS. ON-cultures of EGD-e, ST6 and ST391 were diluted to OD_600_ = 0.02 in XAD-4-treated BHI and grown until OD_600_ = 0.35. Cultures were split and treated with subinhibitory concentrations of EPA (2 µg/mL) or vehicle, included as control. Following 3 hours of either vehicle- or FFA-treatment, samples were harvested from each condition for each strain. Samples were centrifuged at 3200 x g at 4 °C for 5 minutes. The supernatants were discarded, and pellets were resuspended and washed once in 5 mL 1 x PBS. The bacterial inoculum was prepared in DMEM + 10% FBS and used to infect the J774A.1 cells with an MOI of 1 (Deshayes et al., 2012). The bacterial inoculum was added to the wells, and the 24-well plate was centrifuged for 10 minutes at 800 x g to induce phagocytosis of the bacteria. The strains were allowed to be taken up by the cells and replicate intracellularly within the J774A.1 cells for 1 hour, while incubated at 37°C and 5% CO_2_. The bacterial inoculum was determined by plating serial dilutions on BHI agar. Following the primary incubation period, J774A.1 cells were washed twice in 500 µL DMEM + 10% FBS and incubated for an additional 2 or 4 hours with 500 µL DMEM+10% FBS containing 50 µg/mL gentamicin, eliminating extracellular bacteria. After the additional incubation times, cells were washed twice with 500 µL 1xPBS, followed by addition of 500 µL 1xPBS+1% Triton X100, which lyses the J774A.1 cells. The amount of viable, intracellular bacteria was determined by plating serial dilutions on BHI agar and enumerated to determine the ability of each strain to survive and replicate within phagocytic cells. As described in (Deshayes et al., 2012), the number of intracellular bacteria depends on the number of bacteria that were initially phagocytosed by the J774A.1 cells. Therefore, the data from each serial dilutions was normalized using an Intracellular Growth Coefficient (% IGC), which is calculated using the following equation:


$$\%\;IGC=\frac{IB_{t=n}-IB_{t=0}}{IB_{t=0}}$$

Where IB_t=n_ is the intracellular bacterial population at t = n, and IB_t=0_ is the initial intracellular bacterial population (Deshayes et al., 2012). All intracellular proliferation assays were performed in at least three biological replicates and statistical analysis was performed using multiple unpaired t-tests with Welch’ correction. All statistical tests were performed using GraphPad Prism. Differences were considered statistically significant at a confidence level of 95% (p < 0.05).

## Results

### Analysis of virulence profiles in outbreak isolates of *Listeria monocytogenes*

**Figure 1:**
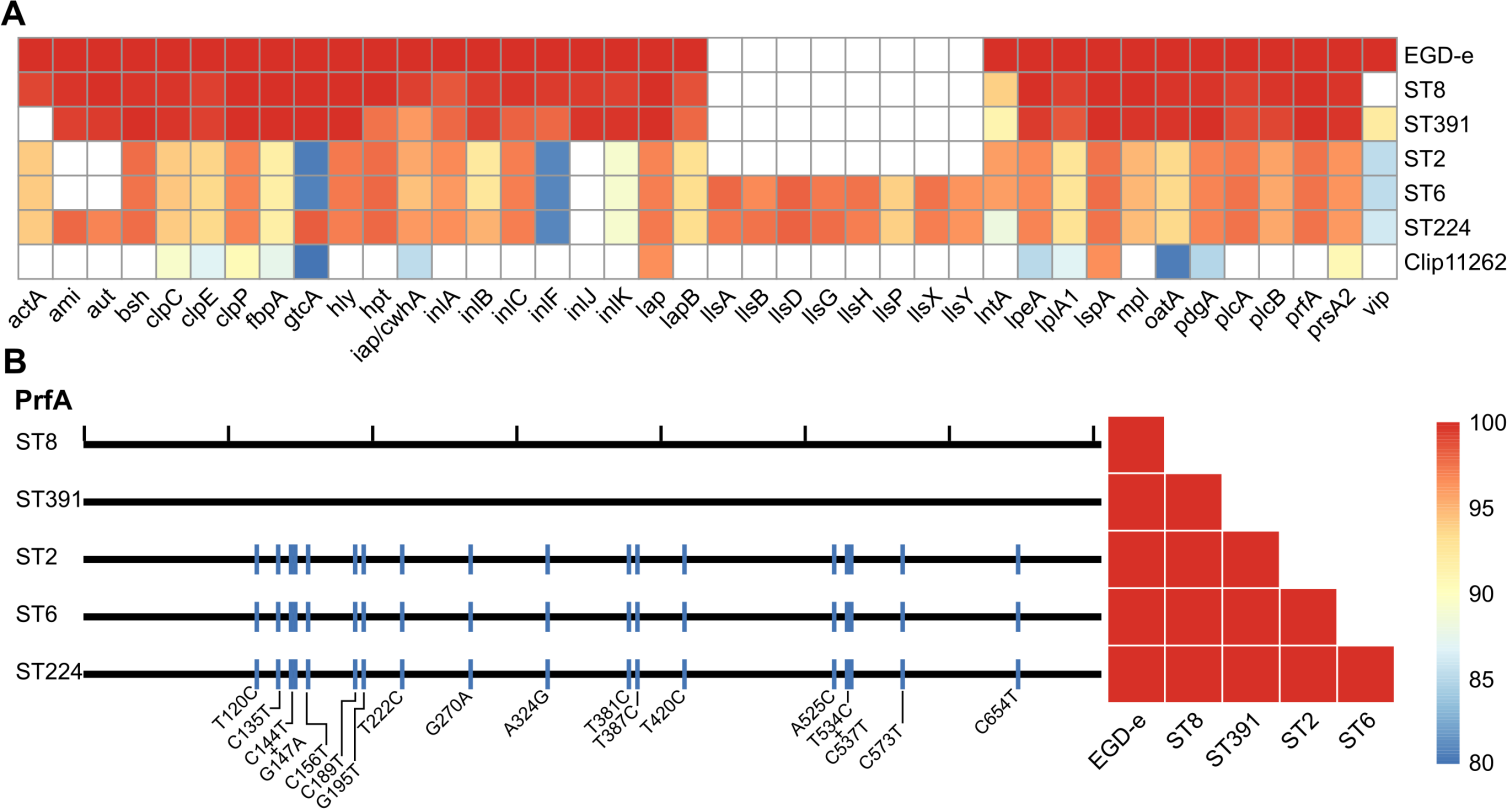
**(A)** Heatmap of *in silico* detected virulence genes for the *L. monocytogenes* laboratory strain EGD-e and clinically relevant isolates (ST8, ST391, ST2, ST6, and ST224). A non-pathogenic *L. innocua* strain, Clip11262, is included as a control. Colored boxes indicate percent identity of identified virulence genes with a minimum of 80% identity and coverage relative to the reference nucleotide sequence; refer to the scale bar for details. White boxes represent either the absence of the gene or sequences with less than 80% identity and coverage relative to the reference nucleotide sequence. **(B)** Left panel: Comparison of the nucleotide sequence of the master transcriptional regulator of virulence, PrfA, between the reference genome (EGD-e) and the five *L. monocytogenes* isolates (ST8, ST391, ST2, ST6, and ST224). Black ticks on the top horizontal line correspond to 100 nucleotides; blue lines indicate different SNPs (single nucleotide polymorphisms) present in the nucleotide sequences of the clinically relevant isolates compared to the reference nucleotide sequence, with the specific changes marked below. Right panel: Comparison of the amino acid sequence of PrfA between EGD-e and the clinically relevant strains (ST8, ST391, ST2, ST6, and ST224).

**Figure 2:**
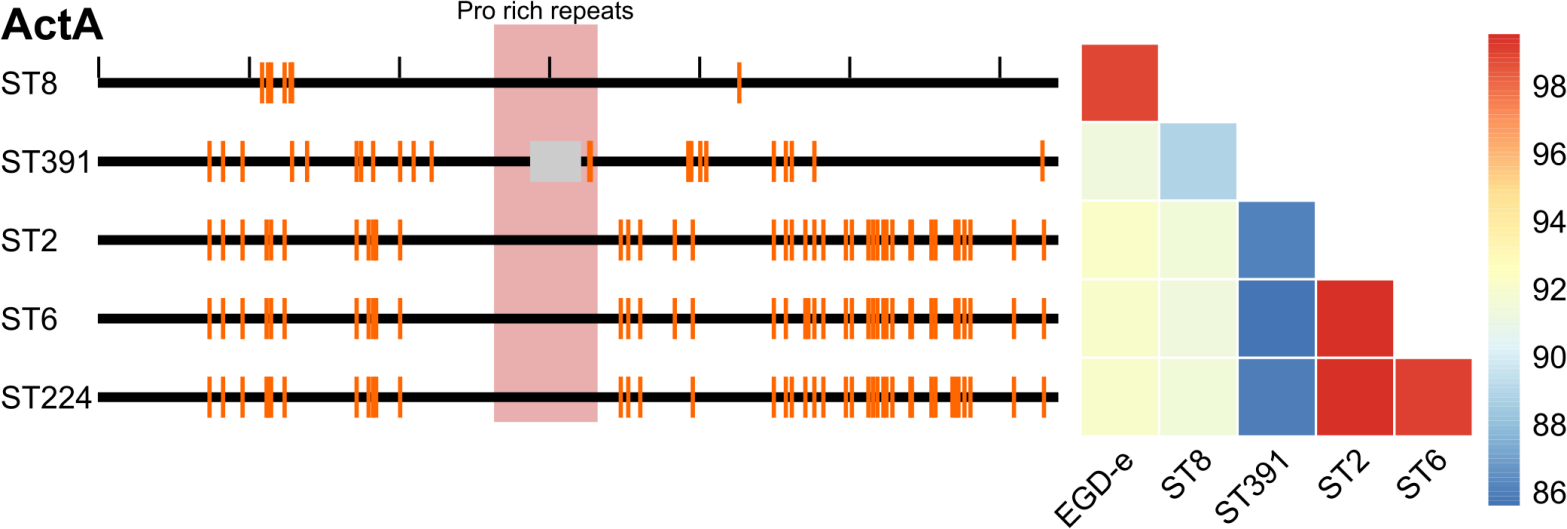
Left panel: Comparison of the nucleotide sequence of the virulence gene *actA* between the reference genome (EGD-e) and five clinically relevant *L. monocytogenes* isolates (ST8, ST391, ST2, ST6, and ST224). Black ticks along the top horizontal line corresponds to 100 nucleotides. Orange lines denote SNPs in the nucleotide sequences compared to the reference genome. The proline-rich repeats encoded within the *actA* gene are highlighted in red, while the grey box indicates an in-frame deletion within *actA* specific to the ST391 isolate. Right panel: Comparison of the amino acid sequence of ActA between the laboratory strain (EGD-e) and clinically relevant isolates (ST8, ST391, ST2, ST6, and ST224). Amino acid sequences are color-coded according to identity, with the color legend indicating that blue represents the least similar sequences and red represents the most similar sequences.

The genomes of five Danish clinically relevant *L. monocytogenes* isolates, ST2 (CC2, serovar 4b), ST6 (CC6, serovar 4b), ST8 (CC8, serovar 2a), ST224 (CC224, serovar 2b), and ST391 (CC89, serovar 2a) were sequenced and analyzed bioinformatically with respect to their virulence profiles. Virulence genes of the five isolates were detected *in silico* using Abricate and compared to the reference *L. monocytogenes* genome of the laboratory strain, EGD-e, as well as the non-pathogenic *Listeria innocua* reference strain Clip11262 (**Figure 1A**). The *in silico* detection of virulence genes required a minimum of 80% identity and coverage to the reference nucleotide sequence. As expected, EGD-e, along with ST2, ST6, ST8, and ST224, all carry key virulence genes necessary for successful host infection, including the LIPI-1 virulence genes (i.e., *prfA, plcA, hly, mpl, actA*, and *plcB*) in addition to the internalins, *inlA, inlB*, and *inlC*, and the hexose phosphate transporter, *hpt*. The *L. innocua* strain Clip11262 does not carry any of these virulence genes (**Figure 1A**). Interestingly, ST391 appears to lack the *actA* gene with 80% identity and coverage compared to that of the EGD-e strain (**Figure 1A**). To further investigate the apparent dissimilarity or potential absence of this virulence gene in ST391, a manual inspection of *actA* was performed, followed by nucleotide and amino acid sequence comparisons among the six strains (**Figure 2**). Several SNPs were observed that differentiate the outbreak isolates from EGD-e (**Figure 2**, left panel). Notably, ST391 contains an in-frame deletion of the nucleotide sequence encoding 35 amino acids within the proline-rich repeats of ActA (**Figure 2**, left panel). Similar analyses were conducted for other essential virulence factors, including InlA, InlB, Hpt, and LLO (see **Supplementary Figure S1**), confirming that all six strains possess hallmark virulence factors essential for the pathogenesis of *L. monocytogenes*.

As predicted, EGD-e and the outbreak strains harbor the *prfA* gene, which encodes the master regulator of virulence (**Figures 1A, B**). Nucleotide alignments of the *prfA* genes revealed that the sequences of the lineage II strains (EGD-e, ST8, and ST391) are identical (**Figure 1B**, left panel), thus also resulting in identical amino acid sequences (**Figure 1B**, right panel). In contrast, when comparing the nucleotide sequences of the *prfA* gene in EGD-e and the lineage I strains (ST2, ST6, and ST224), several single nucleotide polymorphisms (SNPs) were identified (**Figure 1B**, left panel). However, these nucleotide differences do not translate into variations at protein level (**Figure 1B**, right panel). Consequently, all six strains encode an identical PrfA protein.

Two lineage I isolates, ST6 and ST224, harbor the LIPI-3 gene cluster (i.e., *IlsA, IlsG, IlsH, IlsX, IlsB, IlsY, IlsD*, and *IlsP*, **Figure 1A**), which is known to be present in a subset of hypervirulent lineage I strains of *L. monocytogenes* (Cotter et al., 2008; Meza-Torres et al., 2021). This observation aligns with the understanding that certain lineage I strains exhibit increased virulence compared to lineage II strains. The lineage I strain ST2, however, does not appear to carry the LIPI-3 gene cluster.

### Long-chain polyunsaturated fatty acids inhibit growth of *L. monocytogenes* outbreak strains

To evaluate the antimicrobial properties of selected long-chain FFAs against EGD-e and the outbreak isolates, the strains were exposed to increasing concentrations of FFAs known for their antimicrobial and/or anti-virulent properties (Sternkopf Lillebaek et al., 2017; Dos Santos et al., 2020). The long-chain FFAs selected for this study were the omega-3 fatty acid eicosapentaenoic acid (EPA, C20:5), and four FFAs of the C18-series: γ-linolenic acid (GLA; C18:3), linoleic acid (LNA; C18:2), oleic acid (OA; C18:1), and stearic acid (SA; C18:0). In BHI medium, growth patterns of all six *L. monocytogenes* strains were comparable, indicating that they are equally capable of proliferating under routine laboratory conditions (**Supplementary Figure S2**).

**Figure 3:**
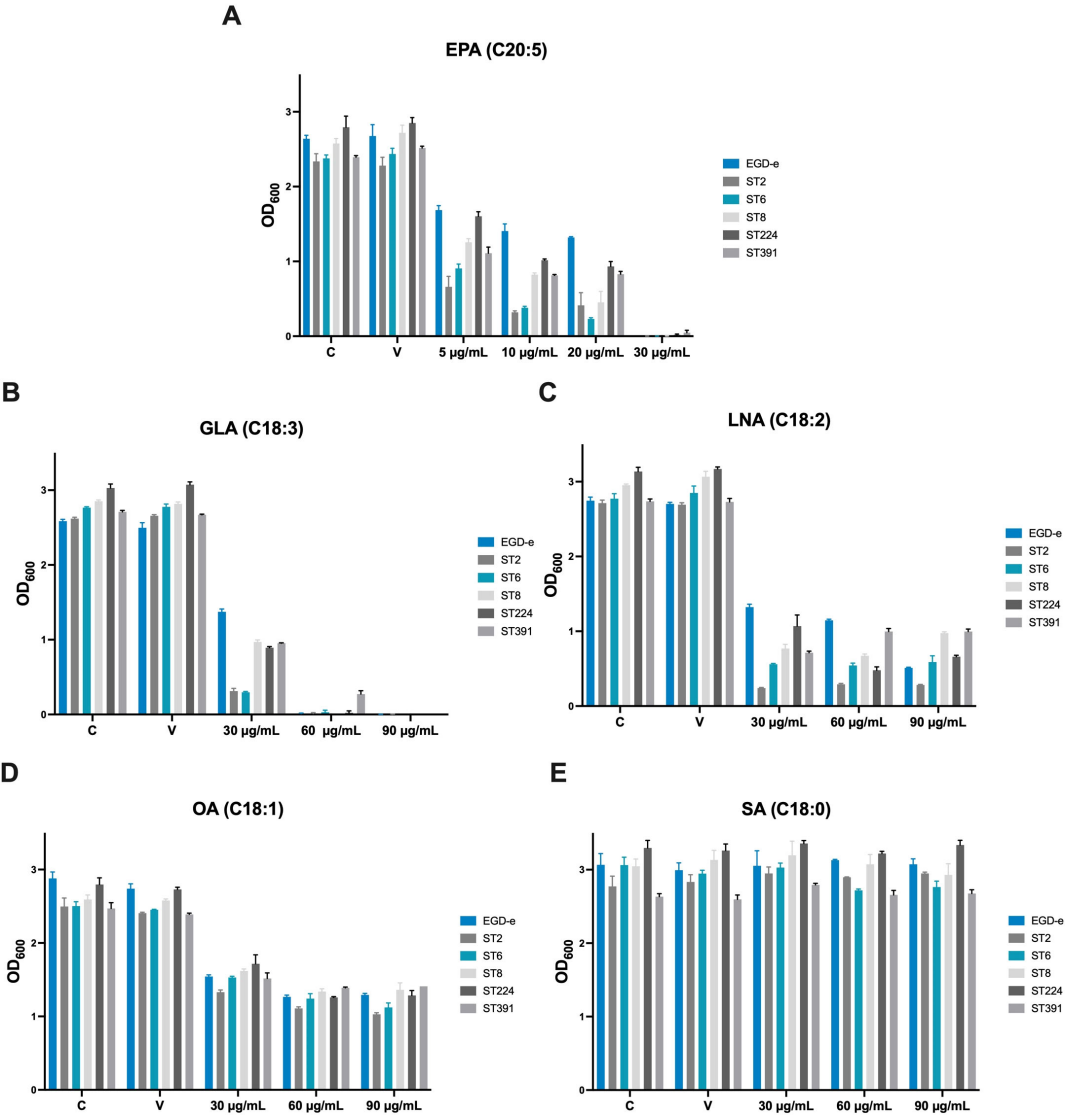
Growth of EGD-e and clinically relevant isolates (ST2, ST6, ST8, ST224, and ST391) in the presence of **(A)** EPA, **(B)** GLA, **(C)** LNA, **(D)** OA, or **(E)** SA. Overnight cultures of the six strains were diluted to an OD_600_ of 0.0002 in BHI medium and subjected to growth in the presence of increasing concentrations of FFAs (5-30 µg/mL EPA or 30-90 µg/mL GLA, LNA, OA, or SA). An untreated culture, labelled “C”, as well as a vehicle-treated culture, labelled “V”, were included as controls. Growth was measured after 20 hours of incubation. The inhibitory concentration (IC) was defined as the lowest concentration of FFA with an OD_600_ of ≤0.1. Results represent the mean of three biological replicates, with error bars displaying the standard deviation among replicates.

The inhibitory concentration (IC) of each FFA was recorded after 20 h of growth. In our previous work, we found that exposure to the omega-3 fatty acid EPA (C20:5) inhibited the growth of *L. monocytogenes* laboratory strains at a concentration of 30 µg/mL, and similar inhibition patterns were observed here for EGD-e and the clinically relevant isolates (**Figure 3**; IC values: 30 µg/mL) (Thomasen et al., 2022a). The C18-series FFA GLA (C18:3) is known to exhibit antimicrobial effects against laboratory strains of *L. monocytogenes*, while LNA (C18:2) and OA (C18:1) are non-antimicrobial but possess anti-virulent properties (Sternkopf Lillebaek et al., 2017; Dos Santos et al., 2020). The saturated counterpart SA (C18:0), however, is neither antimicrobial nor anti-virulent (Sternkopf Lillebaek et al., 2017; Dos Santos et al., 2020). In line with this, growth of EGD-e, ST2, ST6, ST8, and ST224 was completely inhibited at 60 µg/mL GLA, while ST391 exhibited slightly greater tolerance to GLA (**Figure 3**; IC value: 90 µg/mL). In contrast, LNA, OA, and SA did not exhibit antimicrobial activity against any of the strains, as evidenced by the growth of all six strains at the highest concentrations tested (**Figure 3**; IC values >90 µg/mL). Altogether, these results suggest that specific long-chain polyunsaturated FFAs, known for their antimicrobial properties against laboratory strains, are also effective in inhibiting the growth of *L. monocytogenes* strains of clinical relevance.

**Figure 4:**
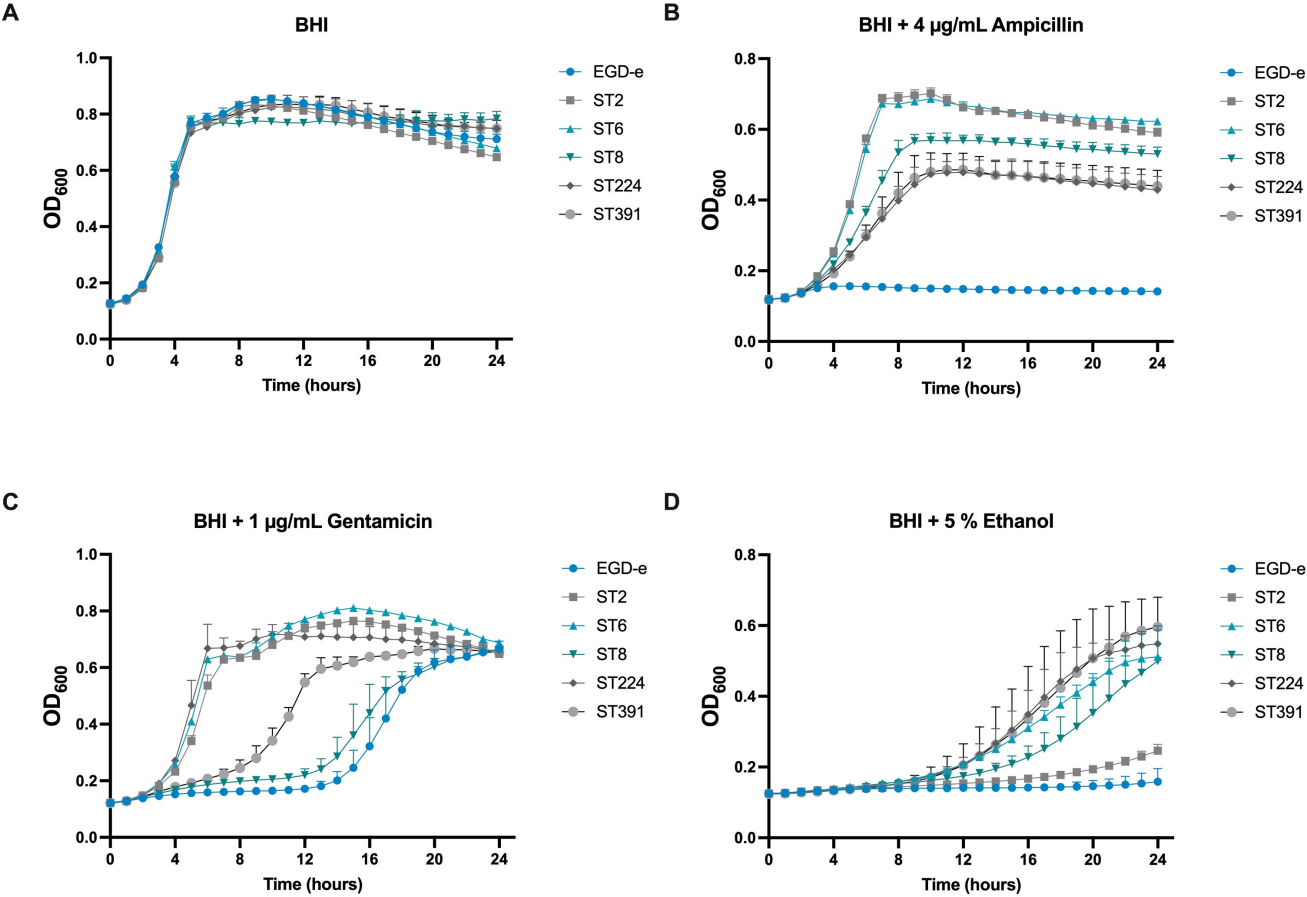
Growth of EGD-e and clinically relevant isolates (ST2, ST6, ST8, ST224, and ST391) in the presence of selected concentrations of stress agents. **(A)** Growth in BHI under non-stressed conditions (BHI). **(B)** Growth in the presence of 4 µg/mL ampicillin, **(C)** 1 µg/mL gentamicin, or **(D)** 5% ethanol. Overnight cultures were diluted to an OD_600_ of 0.005 in BHI medium supplemented with varying concentrations of the stress agents. Growth was monitored over 24 hours. Results represent the mean of three biological replicates, with error bars displaying the standard deviation among replicates.

In addition to FFAs, we aimed to assess the tolerance of the outbreak isolates to other stressors. To this end, the growth of EGD-e and the outbreak strains was monitored under conditions of ethanol stress and antibiotic exposure. Ethanol is frequently used for disinfection, while infections caused by *L. monocytogenes* are typically treated with a combination of conventional antibiotics, specifically β-lactams and aminoglycoside antibiotics (Hof, 2003). Therefore, we chose to examine the growth of EGD-e and the outbreak isolates in the presence of ampicillin or gentamicin. Overall, the outbreak strains exhibited greater tolerance to the stressors compared to EGD-e, particularly in response to ampicillin (**Figure 4**). However, the growth of ST2 in ethanol-supplemented BHI was comparable to that of EGD-e, showing significantly reduced growth relative to the other isolates of clinical relevance (**Figure 4**).

### The omega-3 fatty acid EPA downregulates mRNA levels and cellular protein levels of selected virulence factors in outbreak isolates of *L. monocytogenes*

**Figure 5:**
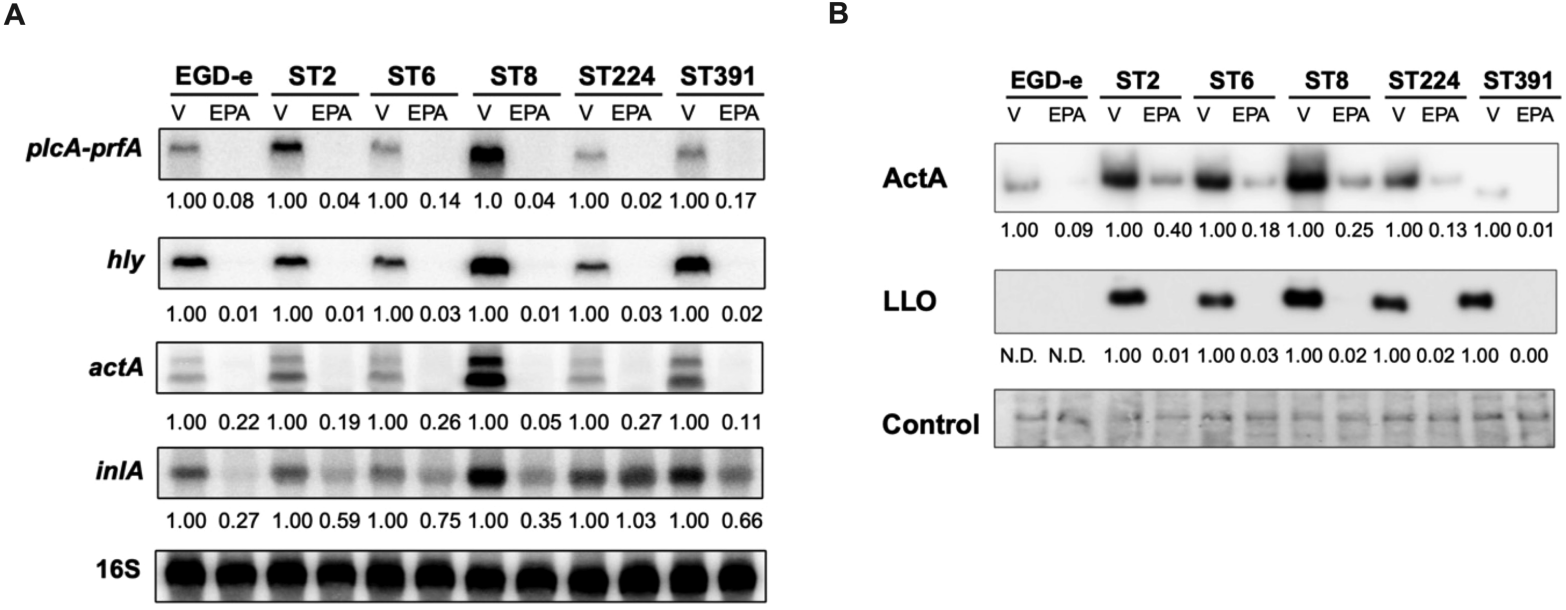
Subinhibitory concentrations of EPA downregulate the mRNA- and cellular protein levels of selected PrfA-regulated virulence genes. **(A)** For Northern blot analysis, EGD-e and five clinically relevant *L. monocytogenes* isolates (ST2, ST6, ST8, ST224, and ST391) were grown to an OD_600_ of 0.35 in XAD-4-treated BHI and subjected to subinhibitory concentrations of EPA (2 µg/mL) for 30 minutes. A vehicle-treated sample, labelled “V”, was included as a control. Following exposure to EPA or control conditions, total RNA was extracted. mRNA levels of *prfA* co-transcribed with *plcA*, as well as *hly, actA*, and *inlA*, were examined using radiolabeled probes (see **Supplementary Table S1**). Relative levels of the transcripts (normalized to 16S rRNA and relative to V) are shown below each lane. **(B)** For Western blot analysis, all strains were grown to an OD_600_ of 0.35 in XAD-4-treated BHI and then exposed to 2 µg/mL EPA for 3 hours. A vehicle-treated sample, labelled “V”, was included as a control. Following exposure to EPA or the vehicle, cells were harvested for Western blot analysis. Cellular protein levels of ActA and LLO were detected by antibodies specific to these proteins. N.D.: not detected. For the loading control, all proteins on the membrane were stained with Coomassie. The relative levels of LLO and ActA (normalized to the loading control and relative to V) are shown below each lane.

The omega-3 fatty acid EPA is known to exert an anti-virulent effect on laboratory strains of *L. monocytogenes* (Sternkopf Lillebaek et al., 2017; Dos Santos et al., 2020). At subinhibitory levels, EPA represses the expression of key virulence factors, including LLO and ActA, in *L. monocytogenes* EGD-e (Sternkopf Lillebaek et al., 2017). This suggests that EPA functions as a signal to suppress the transcription of PrfA-dependent virulence genes. To investigate the impact of EPA on the expression of PrfA-regulated virulence genes in clinically relevant isolates, we conducted Northern blot analyses. The strains were cultured to early exponential phase in BHI medium supplemented with XAD-4, a condition known to enhance the expression of PrfA-regulated virulence genes in EGD-e (Portman et al., 2017). Total RNA was extracted from cultures treated with sub-inhibitory concentrations of EPA or the vehicle as control. Northern blotting was then performed to visualize the mRNA levels of PrfA-regulated virulence genes, including the bicistronic transcript of *plcA*-*prfA*, as well as *hly*, *actA*, and *inlA* (**Figure 5A**).

Vehicle-treated samples of EGD-e and the outbreak isolates exhibited strong expression of the selected PrfA-regulated virulence genes (**Figure 5A**). Exposure to subinhibitory concentrations of EPA rapidly repressed the mRNA levels of *prfA* (co-transcribed with *plcA*) in the EGD-e strain. Notably, the same repression was observed across all five strains of clinical relevance, clearly demonstrating that the anti-virulent properties of EPA are not limited to *L. monocytogenes* laboratory strains. Similarly, EPA-mediated downregulation of the other PrfA-regulated virulence genes tested was observed in all six strains (**Figure 5A**).

Based on these results, we sought to determine whether EPA-mediated repression is also observed at the protein level for PrfA-regulated virulence factors. To this end, we conducted Western blot analyses to analyze the cellular protein levels of ActA and LLO in cultures treated with subinhibitory concentrations of EPA or the vehicle (**Figure 5B**). Under control conditions, EGD-e expresses lower protein levels of ActA and, particularly, LLO relative to the *L. monocytogenes* outbreak strains, making it more challenging to visualize these proteins in EGD-e (**Figure 5B**). This difference in expression is less pronounced at the mRNA level, where *actA* and *hly* expression in EGD-e is clearly detectable under control conditions (**Figure 5A**). ST391, which harbors an in-frame deletion within the *actA* gene (**Figure 1**, **Figure 2**), produces a shorter ActA protein compared to the other strains (**Figure 5B**). Consistent with previous findings (Sternkopf Lillebaek et al., 2017), exposure to EPA markedly reduced the level of ActA expressed in the laboratory strain EGD-e (**Figure 5B**). A similar trend was observed in the outbreak isolates, where a clear reduction in protein levels of ActA and LLO was detected, demonstrating that the anti-virulent effect of EPA extends to clinically relevant strains of *L. monocytogenes* (**Figure 5B**). These findings collectively confirm that, at subinhibitory levels, EPA functions as a signal to suppress the expression of PrfA-regulated virulence factors in *L. monocytogenes* outbreak isolates.

### Exposure to the omega-3 fatty acid, EPA, decreases the invasion of Caco-2 cells by *L. monocytogenes* outbreak isolates

**Figure 6:**
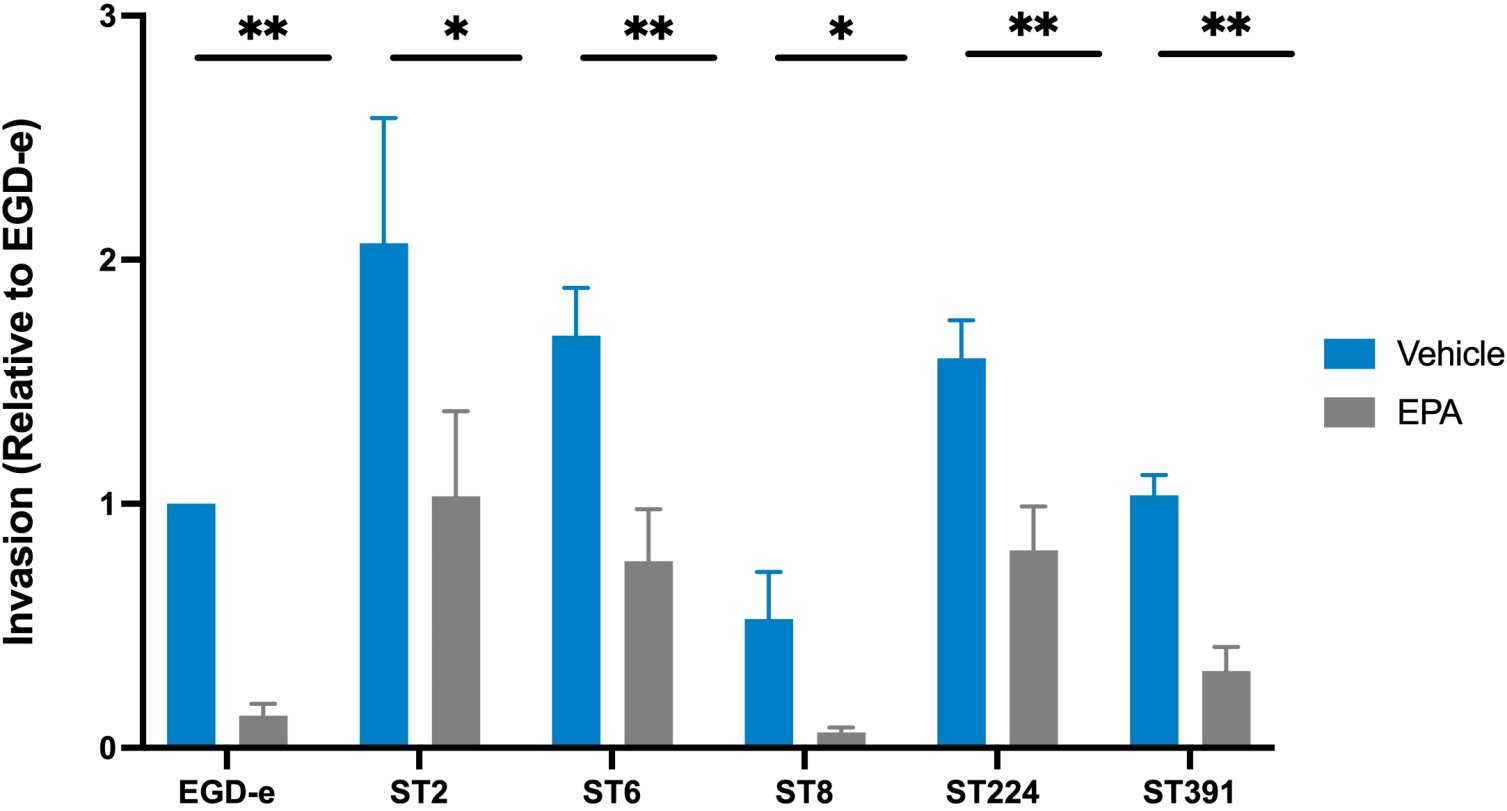
Invasion assay of Caco-2 cells by *L. monocytogenes* EGD-e and five clinically relevant isolates, treated with either ethanol (vehicle) or with subinhibitory concentrations of EPA (2 µg/mL). The bacterial strains infected Caco-2 cells at a multiplicity of infection (MOI) of 100. Data represents the mean values of three biological replicates, with standard deviations between replicates displayed as error bars. Invasion (relative to EGD-e) refers to the invasion percentage for a given strain (vehicle or EPA) relative to the invasion percentage for EGD-e (vehicle). Under control conditions, the invasion percentage for each strain were as follows: EGD-e (0.07%); ST2 (0.19%); ST6 (0.16%); ST8 (0.05%); ST224 (0.14%), and ST391 (0.1%). Upon EPA pre-exposure, invasion was reduced as follows: EGD-e by 5.7-fold, ST2 by 2-fold, ST6 by 2.2-fold, ST8 by 8.32-fold, ST224 by 1.8-fold, and ST391 by 3.2-fold. Statistical analysis was conducted using an unpaired t-test with Welch correction; *p < 0.05, **p < 0.01.

As demonstrated above, exposure to EPA reduces the mRNA- and protein levels of selected PrfA-regulated virulence factors in clinically relevant strains of *L. monocytogenes* (**Figure 5**). Based on these findings, we sought to determine whether pre-exposure to EPA would affect the ability of the outbreak strains to infect host cells. To investigate this, we conducted an invasion assay using the human colonic carcinoma Caco-2 cell line, which models the human intestinal epithelium - the primary site of *L. monocytogenes* infection. Since the infection process relies on the expression of PrfA and its associated virulence factors, we hypothesized that pre-exposure to EPA might reduce the ability of both laboratory strains and outbreak isolates of *L. monocytogenes* to invade Caco-2 cells. To test this hypothesis, EGD-e and the outbreak strains were grown in XAD-4-treated BHI until early exponential phase. At this stage, all six strains were pre-exposed to subinhibitory concentrations of EPA (or the vehicle) for 3 hours, a duration sufficient to visibly reduce the levels of PrfA-regulated virulence factors (**Figure 5B**). The cultures were then harvested and assessed for their ability to invade Caco-2 cells. Following infection, the Caco-2 cells were lysed, intracellular bacteria were enumerated, and the invasion capacity of each strain was determined relative to EGD-e. As shown in **Figure 6**, EGD-e and all examined outbreak isolates were capable of invading Caco-2 cells under control conditions. Importantly, pre-exposure to EPA significantly reduced the ability of all strains to invade Caco-2 cells, with up to an 8-fold reduction in host cell invasion compared to vehicle-treated controls (**Figure 6**).

**Figure 7:**
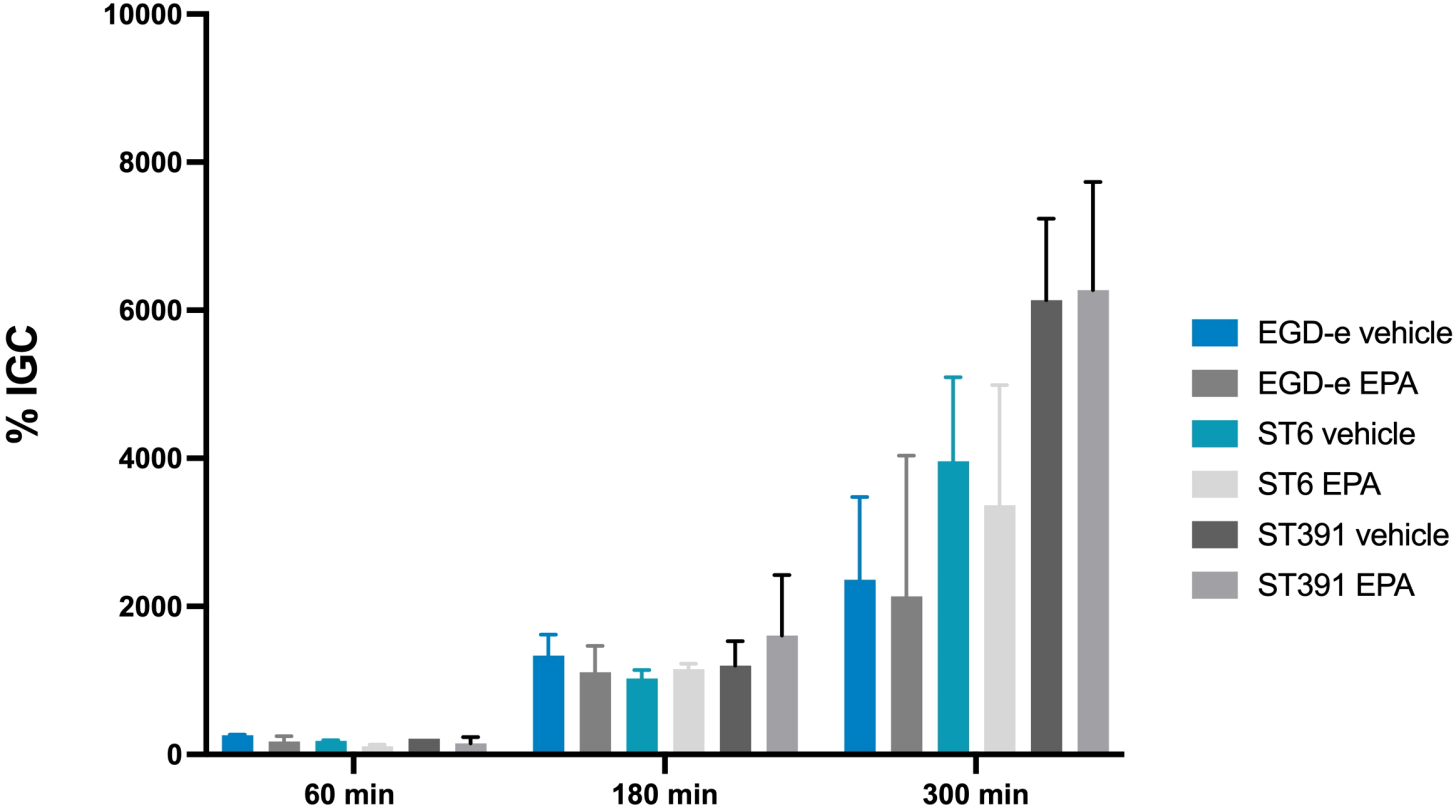
Intracellular replication assay in J774A.1 cells, conducted with *L. monocytogenes* EGD-e and two outbreak isolates (ST6 and ST391). Prior to infection, strains were treated with either ethanol (vehicle control) or subinhibitory concentrations of EPA (2 µg/mL) for 3 hours. The bacterial strains were allowed to replicate intracellularly at a MOI of 1. Data represents the mean of two independent experiments, each performed in triplicate, with standard deviations displayed as error bars. % IGC: Intracellular growth coefficient.

Bacterial survival and replication within phagocytic cells, such as macrophages, are highly dependent on the expression of PrfA-regulated virulence factors, including LLO and ActA. Given that exposure to EPA reduced the expression of these virulence factors (**Figure 5**), we sought to determine whether pre-exposure to this FFA inhibits the ability of *L. monocytogenes* to survive and replicate within phagocytic host cells. To investigate this, we performed an infection assay using the murine macrophage cell line J774A.1. Cultures of EGD-e and two outbreak isolates (the lineage I strain ST6 and the lineage II strain ST391), pre-exposed to subinhibitory concentrations of EPA or a vehicle control, were allowed to replicate within the J774A.1 cells for 60, 180, and 300 minutes. At the indicated time points, cells were lysed, intracellular bacteria were enumerated, and the ability of each strain to replicate intracellularly was determined. Upon treatment with the vehicle control, all three strains demonstrated intracellular growth within the J774A.1 cells (**Figure 7**). Apparently, EPA pre-exposure does not influence the ability of EGD-e, ST6, or ST391 to replicate within phagocytic cells (**Figure 7**). This finding suggests that pre-exposure to EPA induces a short-term repression of PrfA-regulated virulence factors, which impairs the invasion of non-phagocytic cells (**Figure 6**) but does not affect survival and intracellular replication in phagocytic cells (**Figure 7**).

**Figure 8:**
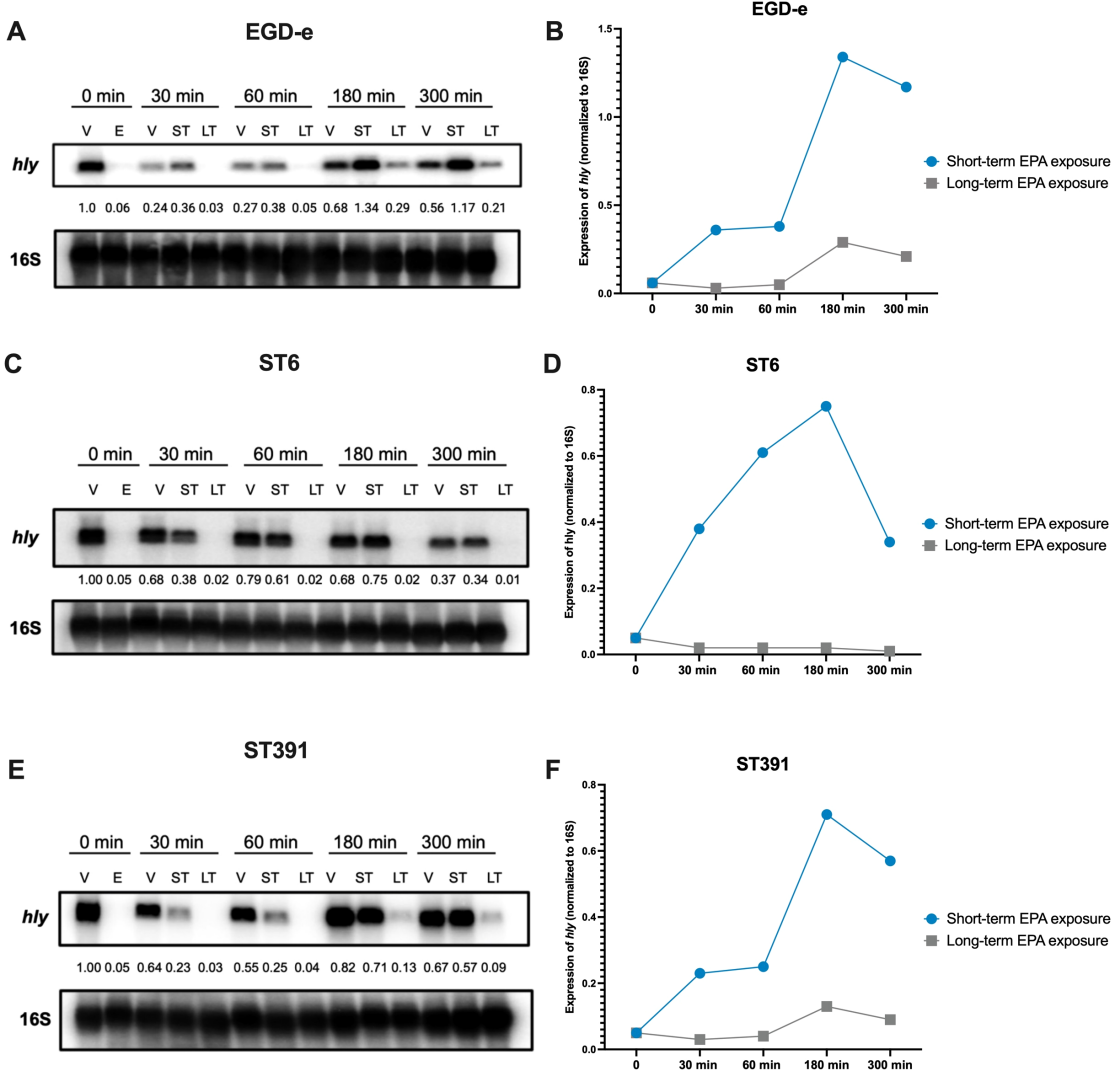
Short-term EPA treatment temporarily reduces the expression of PrfA-regulated virulence gene *hly*. **(A**, **C**, **E)** *L. monocytogenes* EGD-e and two outbreak strains (ST6 and ST391) were grown to an OD_600_ of 0.35 in XAD-4-treated BHI and subjected to the vehicle (V) or subinhibitory concentrations (2 µg/mL) of EPA (E) for 30 minutes. Following this initial pre-exposure, cells were sampled for RNA extraction and Northern blot analysis, corresponding to time 0 min (i.e., samples V and E, respectively). Cultures treated with EPA were then split and exposed to either the vehicle (referred to as “short-term” or “ST” conditions), or continuously exposed to EPA (referred to as “long-term” or “LT” conditions). The vehicle controls (V) were left untreated. At time 30, 60, 180, and 300 minutes, samples were then harvested from the vehicle control (V), EPA short-term (ST), and EPA long-term (LT) exposed cultures. mRNA levels of *hly* were analyzed for each of the three strains using radiolabeled probes. Relative levels of the transcripts (normalized to 16S and relative to V at time 0 minutes) are shown below each lane. **(B**, **D**, **F)** The temporal expression of *hly* mRNA is presented graphically for EPA short-term (ST) and EPA long-term (LT) exposed cultures.

To further examine whether the virulence-inhibitory effect of EPA diminishes over time, we conducted a Northern blot analysis. Total RNA was extracted from cultures of EGD-e, ST6, and ST391 that were pre-exposed to EPA and then grown under control conditions (referred to as short-term exposed cultures). For comparison, total RNA was purified from cultures continuously exposed to EPA (referred to as long-term exposed cultures). RNA levels of *hly* were assessed at 0, 30, 60, 180, and 300 minutes following the initial EPA pre-exposure treatment (**Figures 8A, C, E**). Cultures treated with the vehicle alone throughout the experiment were included as controls (**Figures 8A, C, E**; samples “V”). In strains subjected to short-term EPA exposure, *hly* expression was rapidly upregulated once EPA exposure ceased (**Figures 8A, C, E**; samples “ST”). In contrast, the downregulation of *hly* was more sustained in strains continuously exposed to EPA (**Figures 8A, C, E**; samples “LT”). This pattern was consistent across EGD-e and the outbreak strains when comparing *hly* mRNA levels in short-term and long-term EPA-exposed cultures (**Figures 8B, D, F**). These findings align with the results from the intracellular replication assays in phagocytic cells (**Figure 7**) and support the hypothesis that pre-exposure to EPA induces a transient repression of essential virulence genes, which reverses over time.

### Unsaturated C18 fatty acids downregulate the expression of virulence factors in *L. monocytogenes* outbreak strains and reduce their ability to invade Caco-2 cells

**Figure 9:**
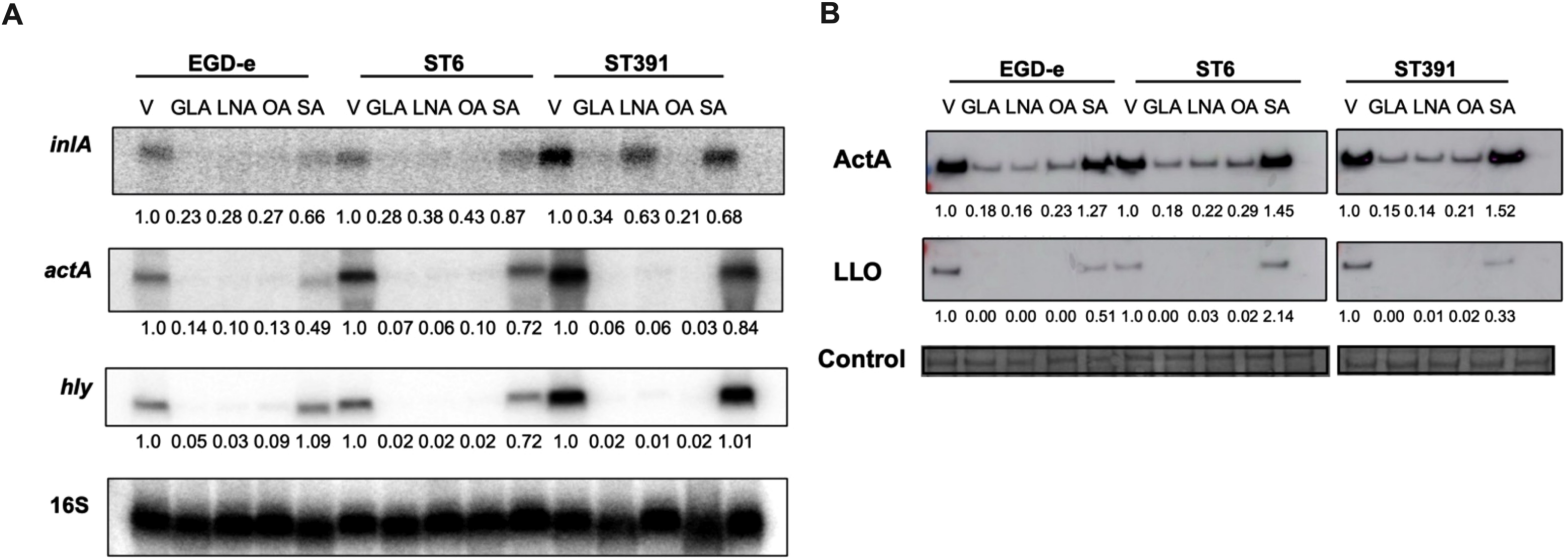
Exposure to unsaturated C18 FFAs downregulates the mRNA and cellular protein levels of PrfA-regulated virulence genes. **(A)** For Northern blot analysis, *L. monocytogenes* strains EGD-e, ST6, and ST391 were grown to an OD_600_ of 0.35 in XAD-4-treated BHI and exposed to subinhibitory concentrations of GLA (2 µg/mL), LNA, OA, or SA (3µg/mL) for 30 minutes. A vehicle-treated sample, labelled “V”, was included as a control. Following exposure to C18 FFAs or control conditions, total RNA was extracted, and Northern blot analysis was performed. mRNA levels of *inlA*, *actA*, and *hly* were examined using radiolabeled probes. Relative levels of the transcripts (normalized to 16S rRNA and relative to V) are shown below each lane. **(B)** For Western blot analysis, *L. monocytogenes* strains EGD-e, ST6, and ST391 were grown to an OD_600_ of 0.35 in BHI medium supplemented with 1% XAD-4. Then, cultures were split and subjected to GLA (2 µg/mL), LNA, OA, or SA (3 µg/mL) for 3 hours. A vehicle-treated sample (V) was included as a control. Following exposure to FFAs or the vehicle, Western blot analysis was performed to visualize cellular proteins levels of ActA and LLO. For the loading control, all proteins on the membrane were stained with Coomassie. The relative levels of LLO and ActA (normalized to the loading control and relative to V) are shown below each lane.

The results obtained so far showed that exposure to the long-chain polyunsaturated FFA, EPA, reduces the expression of PrfA-regulated virulence factors in EGD-e and clinically relevant *L. monocytogenes* strains and decreases their invasion of Caco-2 cells. Following this, we aimed to determine if other long-chain FFAs with varying degrees of unsaturation could exert similar effects. To address this, we analyzed the potential anti-virulent effects of the C18-series FFAs containing three (GLA), two (LNA), one (OA), or zero (SA) double bonds. As shown earlier, high concentrations of GLA exhibited antimicrobial activity against outbreak strains, whereas LNA, OA, and SA did not (**Figure 3**). To investigate their anti-virulent effects, we conducted a Northern blot analysis to evaluate the mRNA levels of selected virulence genes (*inlA*, *actA*, and *hly*) in EGD-e and two outbreak strains, ST6 and ST391, selected as representatives of lineage I and lineage II, respectively. The strains were grown to early exponential phase in XAD-4-treated BHI, and then exposed to subinhibitory concentrations of GLA, LNA, OA, SA or the vehicle as control (**Figure 9A**).

Under control conditions, EGD-e, ST6, and ST391 all displayed strong expression of *inlA*, *actA*, and *hly* (**Figure 9A**). In general, exposure to the unsaturated FFAs GLA, LNA, and OA resulted in a decrease in virulence gene expression, whereas the saturated FFA, SA, had no effect on any of the tested strains (**Figure 9A**). These findings suggest that unsaturated FFAs of the C18-series act as anti-virulence agents against EGD-e as well as *L. monocytogenes* outbreak strains. Following these results, we aimed to determine whether exposure to the C18 FFAs could repress the protein levels of PrfA-dependent virulence factors in EGD-e, ST6, and ST391. To investigate this, cellular proteins were extracted from cultures grown in XAD-4-treated BHI until early exponential phase and exposed to either GLA, LNA, OA, or SA. Protein levels of the chosen virulence factors, ActA and LLO, were detected by Western blot analysis. Exposure to the unsaturated FFAs GLA, LNA, and OA markedly reduced the levels of ActA and LLO in EGD-e, ST6, and ST391, whereas their saturated counterpart, SA, had only minimal effect on the expression of virulence factors (**Figure 9B**). Collectively, these findings demonstrate that exposure to various unsaturated C18 FFAs reduced the levels of PrfA-regulated virulence factors in EGD-e and clinically relevant *L. monocytogenes* strains.

**Figure 10:**
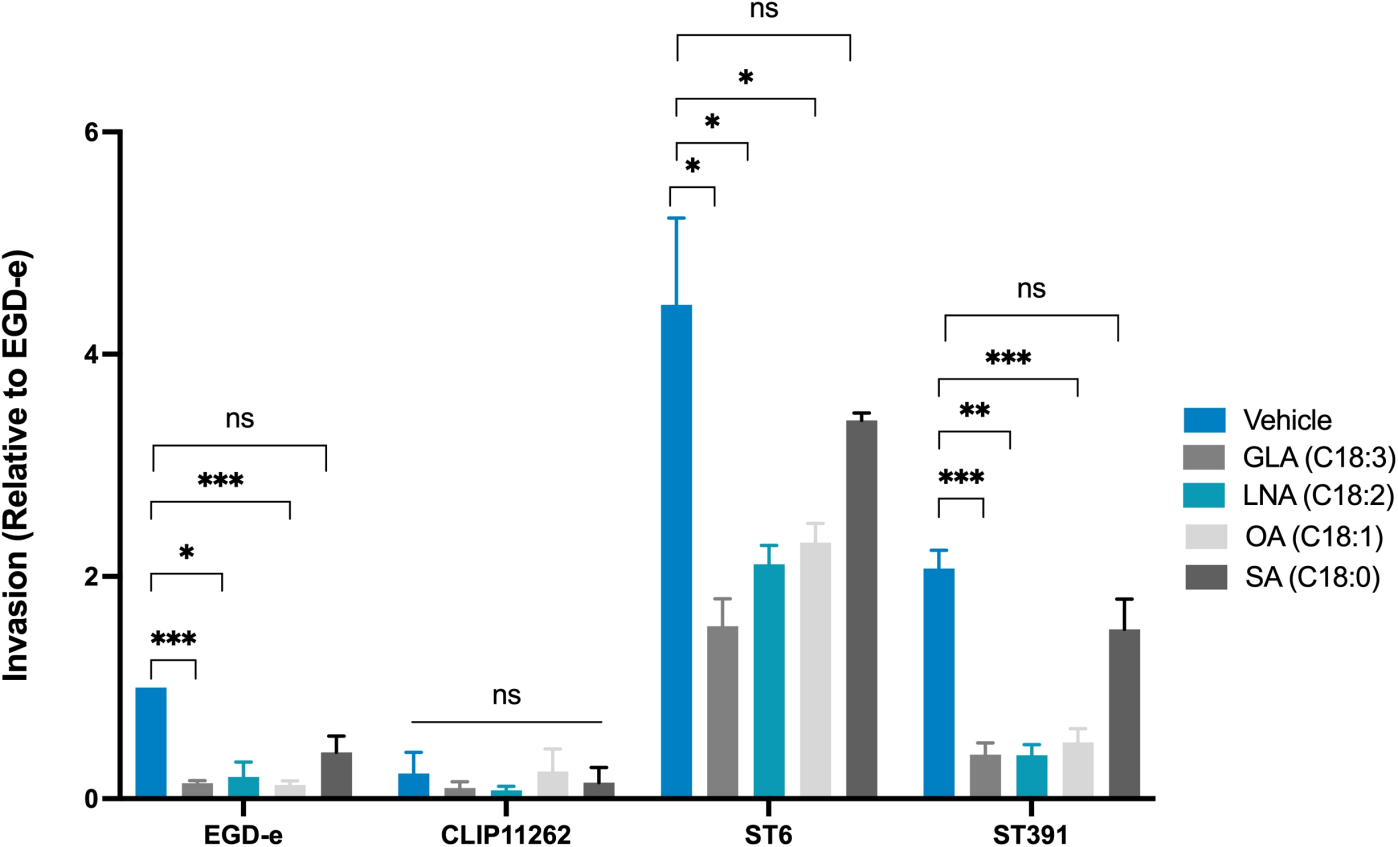
Invasion assay of Caco-2 cells infected with *L. monocytogenes* EGD-e, or two outbreak isolates (ST6 and ST391) treated with either ethanol (vehicle control) or subinhibitory concentrations of GLA (2 µg/mL), LNA, OA, or SA (3 µg/mL). The non-pathogenic strain *L. innocua* Clip11262 was included as a negative control. Bacterial strains were used to infect Caco-2 at a MOI of 100. Data are presented as the mean values of three independent biological replicates, with standard deviations represented as error bars. Statistical significance was determined using an unpaired t-test with Welch’s correction; ns, non-significant, *p < 0.05, **p < 0.01, ***p < 0.001.

As described above, pre-exposing EGD-e and *L. monocytogenes* outbreak strains to EPA reduces their ability to invade Caco-2 cells (**Figure 6**). Therefore, we hypothesized that pre-exposure of *L. monocytogenes* isolates to GLA, LNA, and OA might also inhibit their invasion properties. To investigate this, we conducted an invasion assay using EGD-e, ST6, or ST391 pre-exposed to the vehicle or subinhibitory concentrations of GLA, LNA, OA, or SA. For this experiment, we also included the non-pathogenic *L. innocua* strain Clip11262 as a negative control. As expected, EGD-e and both outbreak strains were capable of invading Caco-2 cells under control conditions (**Figure 10**). Clip11262, however, was not able to infect Caco-2 cells, consistent with the fact that this non-pathogenic strain does not harbor the key virulence factors required for host infection (**Figure 1A**, **Figure 10**). Interestingly, pre-exposure to GLA, LNA, or OA reduced the ability of EGD-e, ST6, and ST391 to infect Caco-2 cells, while pre-exposure to SA did not significantly decrease host cell invasion (**Figure 10**). Altogether, these findings confirm that unsaturated FFAs of the C18-series act as anti-virulence compounds against EGD-e as well as clinically relevant *L. monocytogenes* isolates.

## Discussion

Recent research has shown that long-chain fatty acids act as anti-infective agents against foodborne pathogens, including *L. monocytogenes* (Mitchell and Ellermann, 2022; Borreby et al., 2023). Long-chain fatty acids are abundant in foods and within the gastrointestinal tract, and enteric pathogens appear to use these compounds as signals to adapt to the gastrointestinal environment and establish infections. At high doses, specific long-chain FFAs exert antimicrobial activity, particularly against Gram-positive bacteria, while at low doses, they downregulate the production of virulence factors by targeting sensory proteins or key virulence regulators (Mitchell and Ellermann, 2022; Borreby et al., 2023).

So far, we have used laboratory-type strains as models to analyze the antimicrobial and anti-virulence activities of long-chain FFAs against *L. monocytogenes*. To fully evaluate their potential as anti-infective compounds, however, it is crucial to assess the efficacy of long-chain FFAs against clinically relevant isolates. In this study, we demonstrate that long-chain unsaturated FFAs inhibit the growth and/or virulence potential of selected outbreak strains of *L. monocytogenes*. The outbreak strains encode hallmark PrfA-regulated virulence factors promoting cellular invasion and intracellular replication. ST391, which was responsible for a listeriosis outbreak in Denmark from 2013 to 2015 linked to smoked fish products, encoded a shorter ActA protein relative to that of EGD-e, due to a 35 aa internal deletion. This deletion was also observed in several Norwegian lineage I and II isolates from meat and salmon (Wagner et al., 2022). All strains tested in the present study harbored full-length *inlA* and were capable of invading Caco-2 cells under virulence-inducing conditions (**Figure 6**). Interestingly, the five clinically relevant strains expressed higher levels of ActA and LLO protein compared to the laboratory strain EGD-e (**Figure 5B**). However, these differences in virulence gene expression did not lead to significant differences between EGD-e and the outbreak strains in terms of their ability to infect mammalian cells.

Previously, we observed that *L. monocytogenes* may develop resistance to the antimicrobial effects of FFAs (Thomasen et al., 2022a; Thomasen et al., 2022b). FFA-tolerant strains were readily isolated by serial passage of *L. monocytogenes* in BHI with increasing concentrations of antimicrobial FFAs. Detailed analyses of the FFA-tolerant strains revealed that some are lacking N-acetylglucosamine glycosylation on their wall teichoic acids, whereas others carry mutations within the global regulator CcpA. Furthermore, strains lacking PrfA are tolerant to the antimicrobial activity of FFAs (Sternkopf Lillebaek et al., 2017). In general, the bacterial surface of FFA-tolerant strains is more hydrophilic compared to their parental strains, suggesting that they are better protected against FFA toxicity (Thomasen et al., 2022a; Thomasen et al., 2022b). Since multiple Danish outbreak isolates have been linked to contaminated high-fat fish and delicatessen meats, we considered it relevant to investigate whether they exhibited more tolerance to antimicrobial long-chain FFAs. Although fatty acids in food are mostly found in the form of triglycerides, *L. monocytogenes* is expected to encounter high levels of freely available long-chain FFAs in the intestine, where FFAs are liberated from triglycerides through the enzymatic action of lipases (Borreby et al., 2023). Interestingly, the outbreak strains examined in this study were susceptible to the growth-inhibitory effects of the long-chain polyunsaturated FFAs EPA (C20:5) and GLA (C18:3), suggesting that their responsiveness to antimicrobial FFAs is conserved. The slightly enhanced tolerance of ST391 to GLA (C18:3) could not be attributed to mutations known to confer FFA tolerance (Thomasen et al., 2022a; Thomasen et al., 2022b). Additionally, the outbreak strains demonstrated high sensitivity to the virulence-inhibitory effects of the long-chain unsaturated EPA (C20:5), GLA (C18:3), LNA (C18:2), and OA (C18:1). In laboratory strains, long-chain unsaturated FFAs are known to inhibit PrfA-dependent activation of virulence gene expression (Sternkopf Lillebaek et al., 2017; Dos Santos et al., 2020). PrfA integrates various environmental cues that signal the transition between the saprophytic and pathogenic lifestyles of *L. monocytogenes*. Binding of bacterial- or host-derived glutathione (GSH) is known to induce a conformational change in PrfA that stimulates its ability to bind DNA and activate transcription (Reniere et al., 2015; Hall et al., 2016). Notably, oligopeptides imported via the Opp transport system are proposed to modulate PrfA activity by blocking the GSH-binding site (Krypotou et al., 2019), and other small-molecule inhibitors are predicted to impair PrfA function as well (Good et al., 2016; Tran et al., 2022). Although it is still unclear how *L. monocytogenes* senses and responds to long-chain unsaturated FFAs, our research suggests that they directly interfere with PrfA´s DNA-binding activity (Dos Santos et al., 2020). The outbreak strains analyzed in this study encode a PrfA protein identical to that of the laboratory strains (**Figure 1B**). Consistently, all six strains responded to EPA by downregulating the transcription of PrfA-regulated virulence genes (**Figure 5**). This indicates that the ability to detect and respond to the anti-virulence signaling effects of FFAs is conserved among the strains selected for this study.

We employed cell-based infection assays to investigate how long-chain FFAs influence the ability of *L. monocytogenes* to invade and replicate within mammalian cells. Given the suppressive effect of EPA on virulence gene expression, we hypothesized that this long-chain unsaturated FFA might inhibit *L. monocytogenes* infection. Our results indicate that pre-exposure to EPA significantly reduced the invasive capability of *L. monocytogenes* in non-phagocytic cells (**Figure 6**), while intracellular replication within phagocytic cells remained unaffected (**Figure 7**). These findings suggest that pre-exposure to EPA generates a transient and reversible effect on virulence gene expression in *L. monocytogenes*. Supporting this, we found that *hly* expression quickly resumed upon removal of EPA from bacterial cultures (**Figure 8**), suggesting that continuous exposure to EPA is necessary to sustain its virulence-inhibitory effect. In addition to EPA, pre-treatment of *L. monocytogenes* isolates with unsaturated C18 FFAs reduced their invasive capability, whereas the saturated C18 FFA, SA, had no effect on invasiveness (**Figure 10**). These findings are consistent with previous research, where Caco-2 infection assays were used to evaluate the effect of milk-derived FFAs, including C18 FFAs, on the invasive capacity of *L. monocytogenes* ATCC 7644 (serovar 1/2c) (Petrone et al., 1998). At subinhibitory levels, GLA (C18:3) was particularly effective in preventing invasion by *L. monocytogenes* ATCC 7644 (Petrone et al., 1998). Interestingly, a recent study showed that pre-treatment of Caco-2 monolayers with LNA (C18:2) before infection with an untreated *L. monocytogenes* 10403S derivative (serovar 1/2a) resulted in a decrease in bacterial translocation through the Caco-2 monolayers (Jin et al., 2024). LNA was found to protect tight junctions in Caco-2 monolayers and enhance gut barrier function in mice. Oral pretreatment of mice with LNA prior to infection with *L. monocytogenes* led to significantly decreased loads of the pathogen and attenuated damage to the intestinal epithelial barrier. The study also proposed that the beneficial bacterium *Akkermansia muciniphila* protects against *L. monocytogenes* infection partly by increasing intestinal LNA levels (Jin et al., 2024). Importantly, long-chain FFAs are not metabolized by *L. monocytogenes*, because this pathogen does not encode a functional fatty acid β-oxidation pathway (Glaser et al., 2001; Sauer et al., 2019). Altogether, these findings highlight the potential of long-chain FFAs derived from the diet and/or gut microbiota in preventing *L. monocytogenes* infection.

In summary, this study demonstrates that clinically relevant isolates of *L. monocytogenes* are sensitive to the antimicrobial and anti-virulent activity of long-chain unsaturated FFAs. They are, however, generally more tolerant to antibiotics commonly used to treat *L. monocytogenes* infections compared to the laboratory strain (**Figure 4**). While *L. monocytogenes* is not typically resistant to antibiotics used for treatment, the use of antibiotics in general should be limited and carefully monitored to reduce the emergence of antibiotic resistance. In line with this, alternatives to traditional antibiotics are receiving increasing attention, including compounds that target bacterial virulence. The results obtained in this study confirm that long-chain unsaturated FFAs hold potential as anti-infective compounds against clinically relevant *L. monocytogenes*, including outbreak strains originating from high-fat food products. Understanding how long-chain unsaturated FFAs disarm important pathogens, such as *L. monocytogenes*, of their virulence factors may contribute to the development of new anti-infective strategies for combating bacterial infections in the future.

## Data Availability

The datasets presented in this study can be found in online repositories. The names of the repository/repositories and accession number(s) can be found below: https://www.ncbi.nlm.nih.gov/, PRJNA906001.
