# Supplementary material for: Long-chain unsaturated free fatty acids reduce the host cell invasion of Listeria monocytogenes outbreak strains
Source: Front Cell Infect Microbiol. 2025 Feb 28;15:1542165. doi: 10.3389/fcimb.2025.1542165 (PMC11906444; doi:10.3389/fcimb.2025.1542165)
Supplement: Supplementary file 1 [file DataSheet1.pdf]

Supplementary Material

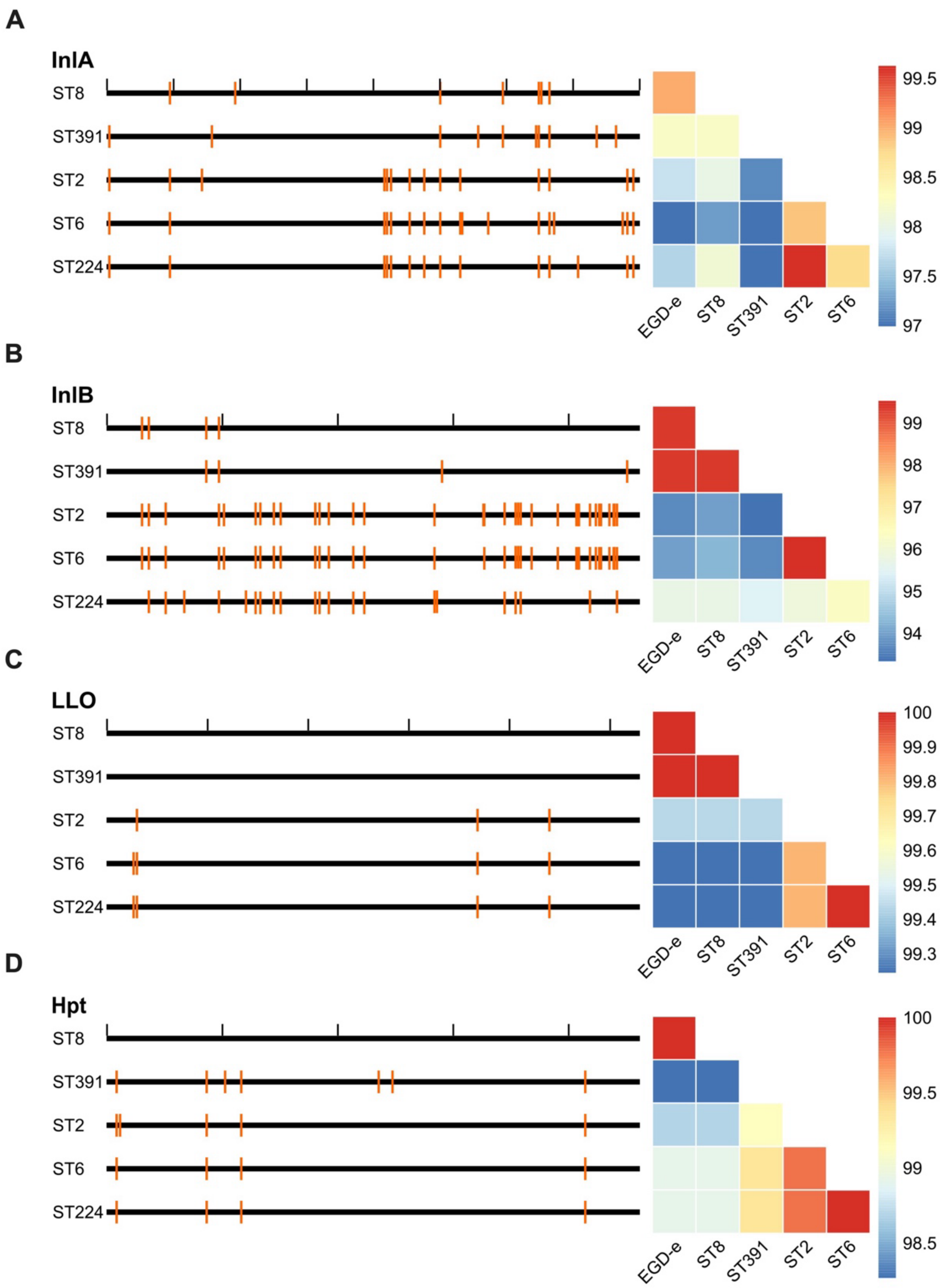

Supplementary Figure S1. Left panels: Comparison of the nucleotide sequence of the virulence genes **(A)** *inlA,* **(B)** *inlB,* **(C)** *hly*, or **(D)** *hpt* between the reference genome (EGD-e) and five *L. monocytogenes* isolates of clinical relevance (ST8, ST391, ST2, ST6, and ST224). Black ticks along the top horizontal line correspond to 100 nucleotides, while orange lines denote SNPs in the nucleotide sequences compared to the reference genome.

Right panels: Comparison of the amino acid sequences of **(A)** InlA, **(B)** InlB, **(C)** LLO, and **(D)** Hpt between the laboratory strain (EGD-e) and five clinically relevant isolates (ST8, ST391, ST2, ST6, and ST224). Amino acid sequences are color-coded according to similarity, with the color legend indicating that blue represents the least similar sequences and red represents the most similar sequences.

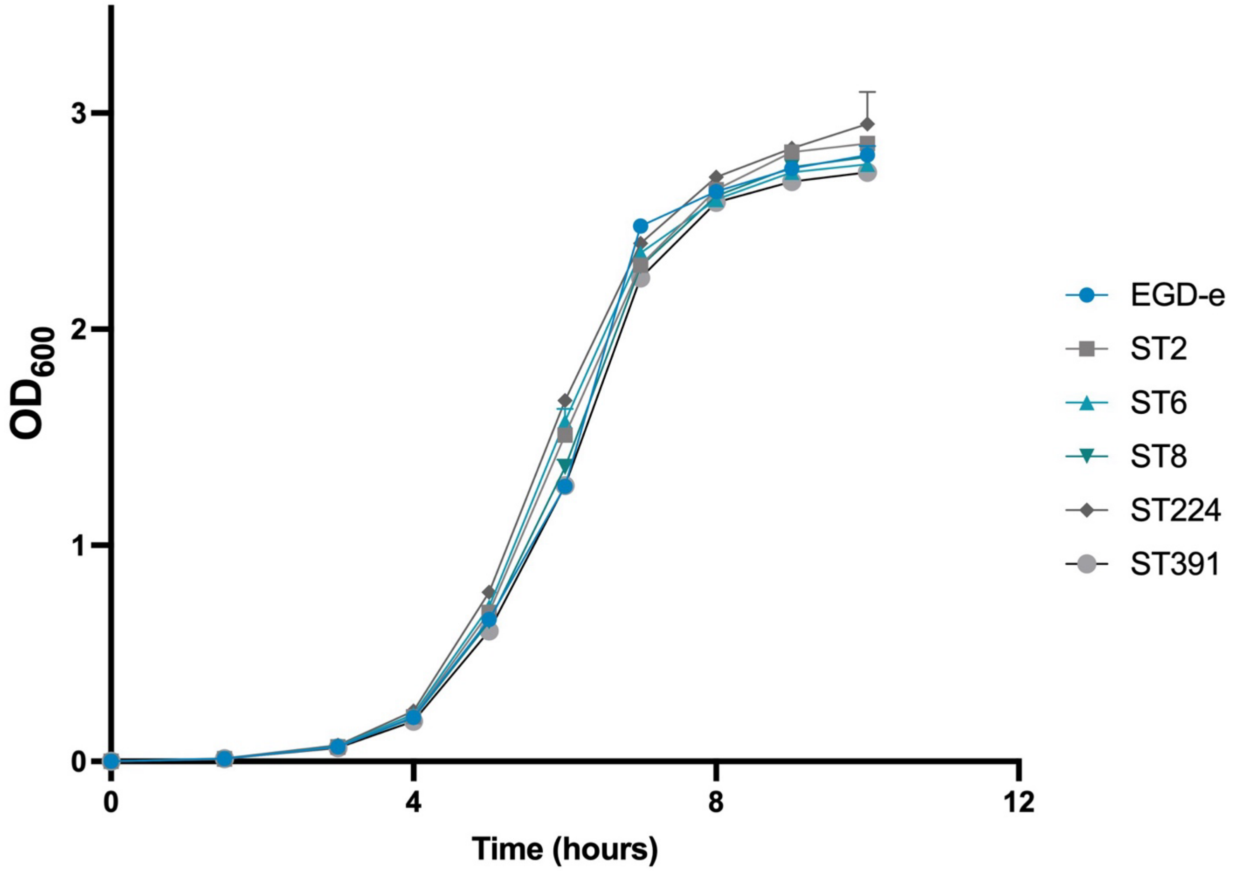

Supplementary Figure S2: Growth of *L. monocytogenes* strains EGD-e, ST2, ST6, ST8, ST224, and ST391 in BHI medium. Overnight cultures of all six strains were diluted to an OD_{600} of 0.002 in BHI medium, and growth was monitored until the stationary phase was reached for all strains. Results represent the mean values of three biological replicates, with error bars displaying the standard deviation among replicates.

Supplementary Table S1. Probes used for Northern blot analysis in this study

**Name**	**Sequence (5’ → 3’)**	**Information**
ActA R	GCTATTAGGTCTGCTTTGTTC	Single-stranded probe for *actA* mRNA
Hly R	CCATCTTTGTAACCTTTTCTTGG	Single-stranded probe for *hly* mRNA
InlA R	ATTTGCGGAAGGTGGTGTAG	Single-stranded probe for *inlA* mRNA
PrfA R	GCTAGACTGTATGAAACTTG	Single-stranded probe for *plcA-prfA* mRNA
16S rRNA	GGCCATTACCCTACCAACTAGCTAATGCAC	Single-stranded probe for 16S rRNA
